# Immune escaping of the novel genotypes of human respiratory syncytial virus based on gene sequence variation

**DOI:** 10.3389/fimmu.2022.1084139

**Published:** 2023-01-10

**Authors:** Xiaohe Zhou, Mingli Jiang, Fengjie Wang, Yuan Qian, Qinwei Song, Yu Sun, Runan Zhu, Fang Wang, Dong Qu, Ling Cao, Lijuan Ma, Yanpeng Xu, Ri De, Linqing Zhao

**Affiliations:** ^1^ Laboratory of Virology, Capital Institute of Pediatrics, Beijing, China; ^2^ Beijing Key Laboratory of Etiology of Viral Diseases in Children, Capital Institute of Pediatrics, Beijing, China; ^3^ Clinical Laboratory, Affiliated Children’s Hospital, Capital Institute of Pediatrics, Beijing, China; ^4^ Intensive Care Unit, Affiliated Children’s Hospital, Capital Institute of Pediatrics, Beijing, China; ^5^ Department of Respiratory, Affiliated Children’s Hospital, Capital Institute of Pediatrics, Beijing, China

**Keywords:** human respiratory syncytial virus, viral genetic differences, antiviral effects, chemokines and cytokines, immune evasion

## Abstract

**Purpose:**

Immune escaping from host herd immunity has been related to changes in viral genomic sequences. The study aimed to understand the diverse immune responses to different subtypes or genotypes of human respiratory syncytial virus (RSV) in pediatric patients.

**Methods:**

The genomic sequences of different subtypes or RSV genotypes, isolated from Beijing patients, were sequenced and systematically analyzed. Specifically, the antiviral effects of Palivizumab and the cross-reactivity of human sera from RSV-positive patients to different subtypes or genotypes of RSV were determined. Then, the level of 38 cytokines and chemokines in respiratory and serum samples from RSV-positive patients was evaluated.

**Results:**

The highest nucleotide and amino acid variations and the secondary and tertiary structure diversities among different subtypes or genotypes of RSV were found in G, especially for genotype ON1 with a 72bp-insertion compared to NA1 in subtype A, while more mutations of F protein were found in the NH-2 terminal, including the antigenic site II, the target of Palivizumab, containing one change N276S. Palivizumab inhibited subtype A with higher efficiency than subtype B and had stronger inhibitory effects on the reference strains than on isolated strains. However, RSV-positive sera had stronger inhibitory effects on the strains in the same subtypes or genotypes of RSV. The level of IFN-α2, IL-1α, and IL-1β in respiratory specimens from patients with NA1 was lower than those with ON1, while there were higher TNFα, IFNγ, IL-1α, and IL-1β in the first serum samples from patients with ON1 compared to those with BA9 of subtype B.

**Conclusions:**

Diverse host immune responses were correlated with differential subtypes and genotypes of RSV in pediatric patients, demonstrating the impact of viral genetics on host immunity.

## 1 Introduction

Human orthopneumovirus, also called human respiratory syncytial virus (RSV), is a common respiratory virus that affects the lungs and respiratory tract, which can lead to hospitalization in severe cases. Importantly, it is the second largest pathogen that causes acute lower respiratory tract infection (ALRTI) deaths in infants and children under the age of 5 years (1). The global burden of virus-associated acute lower respiratory tract infections in 2019 linked RSV to 25.4 million to 44.6 million infections, 2.9 million to 4.6 million hospitalizations, and 15100 to 49100 deaths in hospitalized children under the age of 2 years (2). In infants, RSV infection is the most common cause of bronchiolitis and pneumonia (3). A clear health burden of RSV and high severity of RSV infection were found even in influenza season (4). Moreover, exacerbated inflammatory damage in the respiratory and nervous systems due to RSV infection was found in both pediatric and elderly populations (5, 6). Thus, RSV infection causes a heavy burden on public health and the social economy worldwide.

RSV belongs to the genus *Orthopneumovirus* of the *Pneumoviridae* family (7). It is a single-stranded and negative-sense RNA-enveloped virus with a 15.2 kb genome containing 10 genes that encode 11 proteins, including envelope spikes fusion protein (F), attachment protein (G), and small hydrophobic protein (SH), inner envelope proteins (matrix M, M2-1, and M2-2), non-structural proteins (NS1 and NS2) and ribonucleocapsid complexes phosphoprotein (P) and nucleoprotein (N). There is no proofreading mechanism for the RNA-dependent genome replication of RSV, which enables RSV to rapidly generate single nucleotide polymorphisms and a high rate of mutations (8).

G and F play very important roles in viral entry since they are the major glycoproteins on the surface of virions and thus may serve as ideal and effective binding targets when using neutralizing antibodies. RSV F and G are the only antigens that induce RSV-neutralizing antibody responses. Based on the antigenic differences of F and G, RSV can be divided into two subtypes: A (RSV-A) and B (RSV-B). Currently, many vaccines under development mostly target F since fusion glycoprotein F has a higher degree of conservation (>90%) among RSV strains (9), which is also more immunogenic and cross-protective than glycoprotein G (10). The RSV-neutralizing monoclonal antibody, Palivizumab, appears to target the antigenic site II in the prefusion form of F (1). For G, there are two hypervariable regions, HVR1 and HVR2, of the extracellular region of the highly variable G, separated by a highly conserved region with 13 aa (aa 163–189) (11). RSV A and B subtypes can be further divided into various genotypes due to the high variability of the nucleotide sequences of HVR2. The novel genotypes ON1 (subtype A) and BA9 (subtype B) are becoming the dominant genotypes of RSV worldwide. These two novel genotypes harbor a 72- or 60-nucleotide duplicate insertion in HVR2 of the G gene (12–15).

The interaction and combination between host and viral genetics determine the outcomes of virus-induced disease. It has been shown that minimal changes in RSV and human rhinovirus in the viral genome can affect viral virulence and evade host immunity. These changes favor the evasion of antiviral agents and vaccines (16). To determine the impact of duplicate nucleotide insertions of novel genotypes ON1 and BA9 and other viral genetic changes in different subtypes or genotypes of RSV on host immunity, the genomic sequences of different subtypes/genotypes of RSV were obtained in Beijing, and the antiviral effects of F-specific monoclonal antibodies on RSV were evaluated. Human sera from RSV patients were collected to evaluate the cross-reactivity to different subtypes/genotypes of RSV strains. Finally, 38 chemokines and cytokines in respiratory specimens and sera from children with different subtypes/genotypes of RSV were compared and analyzed. Our results suggested that immune escaping of the novel genotypes of RSV existed, which may be explained by the viral genomic variation, including mutations in antigenic site II of F and duplicate nucleotide insertions of G.

## 2 Materials and methods

### 2.1 Virus stock preparation and measurement of median tissue culture infectious dose

Reference strains Long, A2 and CH18537 were obtained from the Chinese Center for Disease Control and Prevention. Clinical strains (69438, 61397, 3047, 69104, and 86673) were isolated from nasopharyngeal aspirates (NPAs) collected from children diagnosed with lower respiratory tract infections and hospitalized in the Affiliated Children’s Hospital of the Capital Institute of Pediatrics, Beijing, China, for respiratory virus screening using direct immunofluorescence (DFA) (Diagnostic Hybrids, Athens, OH, USA), and then were identified for the subtype and genotype of RSV by reverse-transcription polymerase amplification (RT-PCR) and sequence analysis (15).

RSV strains were propagated in Hep-2 cells (Chinese Academy of Medical Sciences & Peking Union Medical College). Cells were cultured in Modified Eagle Medium supplemented with 2% fetal bovine serum, penicillin (100 U/mL), and streptomycin (100 mg/mL). When the 100% cytopathic effects of RSV strains on Hep-2 cells were observed, the cells were disrupted by freeze/thawing three times, followed by low-speed centrifugation (500 g for 10 min). Aliquots of supernatants were frozen at -70°C. The 50% tissue culture infective dose (TCID_50_) of each RSV strain was determined by infecting Hep-2 cells with serial dilutions on a 96-well plate and calculated by the Reed-Muench method.

### 2.2 Clinical specimens

Nasopharyngeal swabs and serum samples were collected from pediatric patients who met the following criteria (1): diagnosed with “bronchitis”, “bronchiolitis”, or “pneumonia” (2); respiratory specimens positive for RSV tested by DFA (Diagnostic Hybrids, Athens, OH, USA), and further identified for subtype and genotype of RSV by sequence analysis (3, 15). had at least one serum sample: the first was collected on days 0 to 3, and the second was collected on days 5 to 7 after the onset of the disease. Meanwhile, serum samples from healthy children aged 0-5 years were collected as controls.

Nasopharyngeal swabs were pelleted by low-speed centrifugation (500 g for 10 min), then aliquots of clarified supernatants were frozen at -80°C. The sera collected were centrifuged at 500 g for 10 min, then stored at -80°C.

### 2.3 Amplification and sequencing of RSV genomic fragments

Total nucleic acid was extracted from 140 µL of each collected virus strain using the QIAamp MinElute Virus Spin Kit (Qiagen GmbH, Germany) according to the manufacturer’s instructions. To amplify the RSV genomic sequences, 15 contigs covering the whole genome of RSV were amplified by high-fidelity long PCR using Platinum^®^ Taq DNA Polymerase (Invitrogen Life Technologies, Carlsbad, CA, USA) and 15 pairs of primers previously described (17). Some were re-designed based on the genomic sequence of RSV A strain ATCC VR26 (AY911262) and RSV B strain (KF826843). Primers were synthesized by Invitrogen Trading Co., Ltd. (Shanghai, China). Those primers are listed in **Table S1**. All PCR products were sequenced by Sino Geno Max Co., Ltd. (Beijing, China).

### 2.4 Bioinformatics analysis

The phylogenetic trees based on viral genomic or gene sequences were constructed using the neighbor-joining method and maximum composite likelihood model of the MEGA version 6.0 software package (18). ProtParam (https://web.expasy.org/protparam/) was used to predict the composition and molecular weight of the protein, NPS@SOPMA (https://pbil.ibcp.fr/), and five calculation methods (GOR, Levin, dual, PHD and CNRS SOPMA) were used for secondary structure prediction. For the construction of three-dimensional structures of G, F, and SH of RSV-NA1, RSV-ON1, and RSV-BA9, the online SWISS-MODEL service platform (https://swissmodel.expasy.org/), in which the tertiary structural models of RSV F were deposited, was used for F, and alpha fold 2.0 was used for G and SH for there was no tertiary structural model built. PyMOL 2.5.1 was used for visualizing and labeling the 3D model.

### 2.5 Micro-neutralization assays

The F-specific monoclonal antibody, Palivizumab (1mg/mL, received from National Institutes for Food and Drug Control), was diluted to 1:100, 1:200, 1:400, 1:800, 1:1600, 1:3200, and 1:6400 with 2% fetal bovine serum of Modified Eagle Medium. The sera collected were also diluted to 1:10, 1:20, 1:40, 1:80, 1:160, 1:320, and 1:640. Viral strains were diluted to 100 TCID_50_. Add 50 uL of diluted virus and Palivizumab or sera to each 96-well plate, followed by 100 uL of 3×10^5^/mL cell suspension. Cells were routinely propagated in a humidified atmosphere of 5% CO_2_ at 33°C, the cytopathic changes were observed daily, and the results were recorded until the fifth day. IC_50_ was calculated by the Reed-Muench method to indicate the antiviral effect of Palivizumab.

### 2.6 Microarray analysis

38 cytokines and chemokines in respiratory or serum samples were measured using MILLIPLEX^®^ Human Cytokine/Chemokine Magnetic Bead Panel (MERCK, Germany) on the liquid phase micro-array analysis platform (model Luminex100; Luminex, Austin, TX, USA). These included: Fibroblast Growth Factor-2 (FGF-2), Interferon -alpha 2 (IFN-α2), Interferon γ (IFN-γ), Interleukin-10 (IL-10), Interleukin-12 (IL-12), Interleukin-12p40 (IL-12p40), Interleukin-12p70 (IL-12p70), Interleukin-13 (IL-13), Interleukin-15 (IL-15), Interleukin-17A (IL-17A), IL-1 receptor antagonist (IL-1RA), Interleukin-1alpha (IL-1α), Interleukin-1 beta (IL-1β), Interleukin-2 (IL-2), Interleukin-3 (IL-3), Interleukin-4 (IL-4), Interleukin-5 (IL-5), Interleukin-6 (IL-6), Interleukin-7 (IL-7), Interleukin-8 (IL-8), Interleukin-9 (IL-9), Interferon-inducible protein 10 (IP-10), Monocyte chemotactic protein 1 (MCP-1), Monocyte chemotactic protein 3 (MCP-3), Macrophage-derived chemokine (MDC), Macrophage inflammatory protein-1 alpha (MIP-1α), Macrophage inflammatory protein-1 beta (MIP-1β), Transforming growth factor-alpha (TGF-α), Tumor Necrosis Factor-alpha (TNF-α), Tumor Necrosis Factor-beta (TNF-β), Vascular Endothelial Growth Factor (VEGF), Epidermal Growth Factor (EGF), Fms-like tyrosine kinase 3 ligand (Flt-3L), Granulocyte colony-stimulating factor (G-CSF), Granulocyte-macrophage colony-stimulating factor (GM-CSF), Growth Factor (GRO), Eotaxin, and Fractalkine. All samples were repeated in duplicate, and the concentrations were demonstrated as a unit of pg/mL. The plates were analyzed using Luminex xPONENT (Luminex), and the results were analyzed with the Luminex SYNCT Data Analysis Software (Luminex).

### 2.7 Statistical analysis

The average values and 95% CI of the cytokines and chemokines in duplicate samples for each child were calculated by SPSS. The values below the detection limit were assigned a 0 pg/mL concentration. The chi-square (χ2) test and rank sum test were used for statistical analyses by SPSS Statistics 22.0 version (IBM, Armonk, NY, USA). For specimens with small sample sizes, “unpaired” in “Experimental design”, “Use nonparametric test in “Assume Gaussian distribution”, then “Mann Whitney test” in “Choose test” was chosen. *P*-values under 0.05 were considered statistically significant. Spearman’s rank correlation coefficient in SPSS was used to analyze the relationship between two related variables. GraphPad Prism 8.0.1 was used for data visualization.

## 3 Results

### 3.1 Genetic differences in RSV

Two subtypes and several genotypes of RSV were identified by phylogenetic trees based on the HVR2 region of G gene sequences: subtype A (69438-NA1, 61397-ON1, 3047-GA2, Long-GA1, A2-GA1), and subtype B (CH18537-GB2, 86673-BA9, and 69104-BA9) (**Figure S1**). The nucleotide homologies of genomic sequences in this study were 80.5-81.3% between subtypes A and B and >95% within the same subtype (**Table S2**).

For the 11 RSV genes, 66.2-86.2% homology in nucleotide and 46.7-96.4% homology in aa were obtained between subtypes A and B. The highest nucleotide and amino acid variabilities were found in G (66.2-68.5% in nucleotides and 46.7-51.7% in aa), followed by M2-2 (68.6–70.6% in nucleotides and 57.1-60.7% in aa) and SH (76.9-81.0% in nucleotide and 67.2-70.3% in aa).

For protein F, 81.7-82.8% in nucleotide and 89-90.6% in amino acid homology were calculated between subtypes A and B, with more mutations found in the NH-2 terminal of F (**Figure S2**). Antigenic site II of F (aa 254-277), as the target of Palivizumab, contains one amino acid change N276S, with N in strains long, A2, and 3047 (GA2) and S in strains 61397 (ON1), 69438 (NA1), CH18537 (GB2), 86673 (BA9), and 69104 (BA9). There were several changes in antigenic site φ, including D220N, K201N, and K209Q from subtype A to subtype B, and G66E/K (G in long, K in A2, and E in others). There were two changes on the antigenic site V, L172Q (L in subtype A, Q in subtype B) and S173 L (S in subtype A, L in subtype B).

Isolated strains 61397 (ON1) and 69438 (NA1) shared 99% homology in nucleotide and 99.5% in aa in the F region, and 92.3% in nucleotide and 95.6% in aa in G. Strain 61397 (ON1) had a 72-nucleotide (nt) insertion in the HVR2 of G compared with 69438 (NA1) (**Figure S3**).

For strains 69104 (BA9) and 86673 (BA9), a 60nt duplicate insertion was found in the HVR2 of G (**Figure S4**). A 72-nt-insertion in strain 61397 (ON1) and a 60-nt-insertion in strains 69104 (BA9) and 86673 (BA9) in G genes increased the number of aa and molecular weight (**Table S3**).

The secondary structures of SH and F were an α-helix, accounting for 42.19% (SH) and 38.15% (F) in genotypes ON1 and NA1 and 60% (SH) and 39.2% (F) in genotype BA9. The secondary structure of G is an irregular curl, accounting for 55.14%, 56.23%, and 64.63% in genotypes ON1, NA1, and BA9, respectively (**Figure S5**).

**Figure 1:**
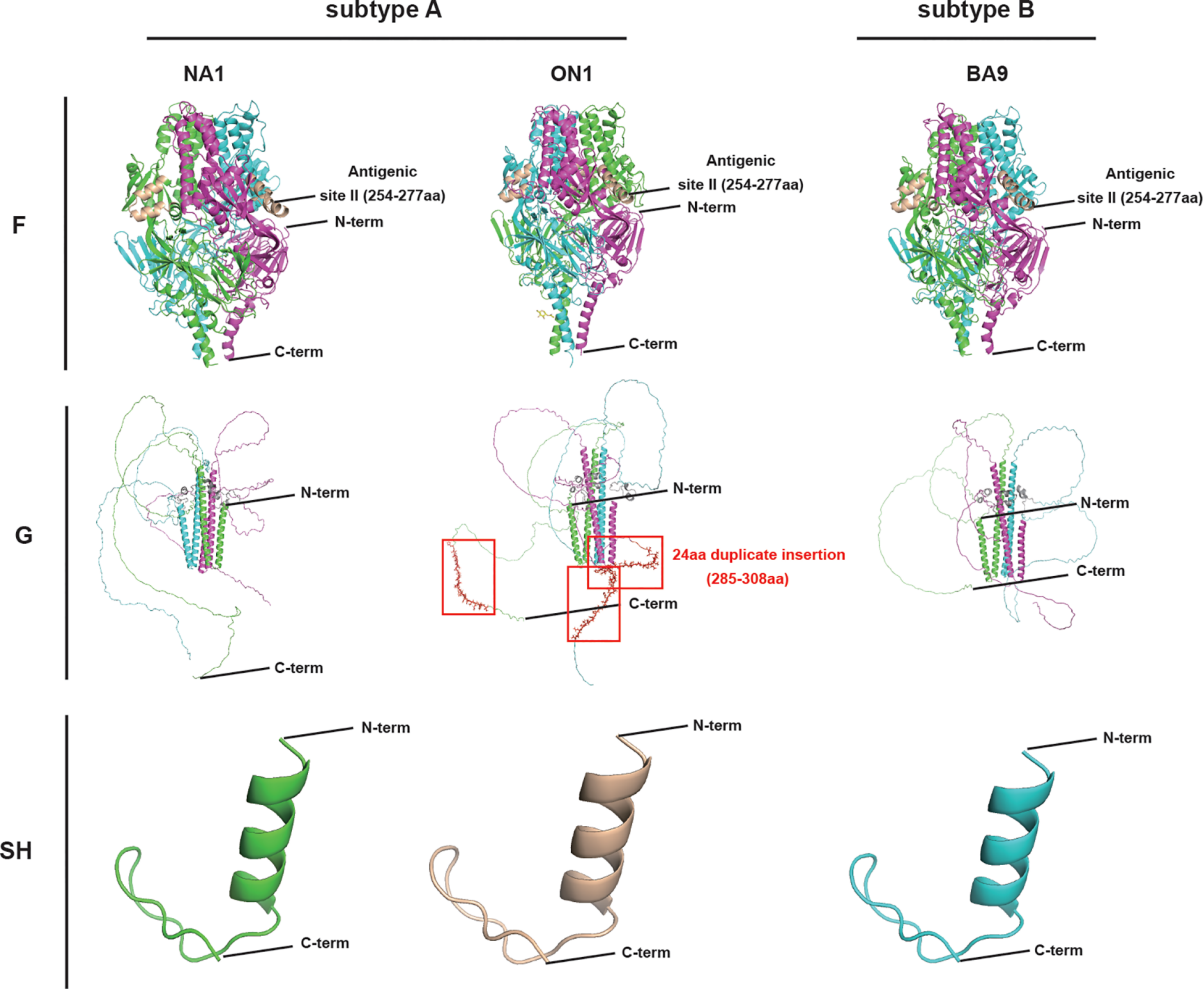
The major surface glycoproteins F, G, and SH tertiary structures of RSV genotypes ON1, NA1, and BA9. In the trimer structure of G and F, each monomer was represented by one color(shown in pink, blue and green). SH of RSV-NA1 (green), RSV-ON1(wheat), and RSV-BA9 (blue) were displayed in the monomer. N-term: N-terminus; C-term: C-terminus; Antigenic site II (254-277 aa) of F: specific sites to Palivizumab. 24 aa duplicate insertion: the most significant structural changes in the HVR2 of G are shown in red boxes.

The results of tertiary structure prediction and three-dimensional (3D) model construction revealed a high similarity in SH between subtypes A and B, whereas a higher similarity in F was found between genotypes ON1 and BA9 than that between ON1 and NA1. However, G’s highest diversity was found among genotypes ON1, NA1, and BA9 (**Figure 1**).

### 3.2 Antiviral effects of Palivizumab to RSV *in vitro*

Palivizumab is an RSV-specific monoclonal antibody, the specific inhibitor targeting the antigenic site II of RSV F. To compare the differences in immunoreactivity of different subtypes or genotypes of RSV to Palivizumab, we evaluated the neutralization effect of Palivizumab on RSV by micro-neutralization assays. 100 TCID_50_ was the virus titer used in the procedure, and Palivizumab was diluted from the maximum non-toxic concentration of 1:200. As shown in **Table 1**, the IC_50_ of Palivizumab to subtype A reference strain Long (GA1) (0.234 μg/mL) was the lowest, followed by the subtype B reference strain CH18537 (GB2) (0.469 μg/mL). In addition, among the local strains of subtype A, the IC_50_ of Palivizumab to 69438 (NA1) (1.875 μg/mL) was significantly higher than that to 61397 (ON1) (0.938 μg/mL) and 3047 (GA2) (0.938 μg/mL).

**Table 1:** The IC_50_ (μg/mL) of Palivizumab to different subtypes and genotypes of hRSV strains.

Antibody	IC_50_ to strains of subtype A				IC_50_ to strains of subtype B		Average of IC_50_	
	Long (GA1)	61397 (ON1)	69438 (NA1)	3047 (GA2)	69104 (BA9)	CH18537 (GB2)	subtype A	subtype B
Palivizumab	0.234	0.938	1.875	0.938	1.875	0.469	0.996	1.172

### 3.3 Cross-reactivity of human sera to RSV

To further determine whether there are differences in the antigenicity of different subtypes or genotypes of RSV, we performed cross-reactivity assays using human sera from different subtypes or genotype RSV-positive patients. As shown in **Table 2**, serum from a patient positive for RSV ON1 (93158) showed strong antiviral ability against the subtype A and B reference strains, as well as the subtype A isolated strains (IC_50_ ≤ 1:198) but weak antiviral activity against the subtype B isolated strain 86673 (BA9) (IC_50_ 1:52). In addition, the antiviral activities of serum from patient positive for RSV NA1 (22190) against subtype A and B reference strains were similar (IC_50_ 1:123), but the antiviral activity against subtype A isolate was significantly better than that of the subtype B isolated strain 86673 (BA9) (IC_50_ 1:87). Meanwhile, serum from patient positive for RSV BA9 (92991) showed stronger antiviral activity to subtype B than to subtype A, with the strongest antiviral activity to isolated strain 86673 (BA9) (IC_50_ 1:276), and the weakest activity to isolated strain 93158 (ON1) (IC_50_ 1:60).

**Table 2:** The IC_50_ of sera from patients with different genotypes of hRSV strains.

Serum	IC_50_ to strains of subtype A					IC_50_ to strains of subtype B		Average of IC_50_	
	Long (GA1)	A2 (GA1)	61397 (ON1)	3047 (GA2)	5540 (NA1)	86673 (BA9)	CH18537 (GB2)	subtype A	subtype B
93158 (ON1)	1:241	1:110	1:417	1:247	1:198	1:316	1:52	1:204	1:129
22190 (NA1)	1:123	1:178	1:251	1:115	1:468	1:87	1:123	1:173	1:105
92991 (BA9)	1:117	1:132	1:60	1:132	1:96	1:276	1:263	1:98	1:269

### 3.4 Diverse immune responses to different subtypes or genotypes of RSV *in vivo*

To evaluate the differences in immune responses to different subtypes or genotypes of RSV, respiratory and serum samples were collected from enrolled patients positive for RSV according to the criteria. There were 40 respiratory specimens genotyped to ON1, BA9, or NA1, including 15 genotyped to ON1, with 15 serum samples collected within 0~3 days (the first serum group) and 14 within 5~7 days after the onset of disease (the second serum group), 17 genotyped to BA9 with 17 serum samples collected within 0~3 days (the first serum group) and 8 within 5~7 days (the second serum group) after the onset of disease, and 18 genotyped to NA1 with no serum samples. In addition, serum samples were collected from 12 healthy children as a control.

**Figure 2:**
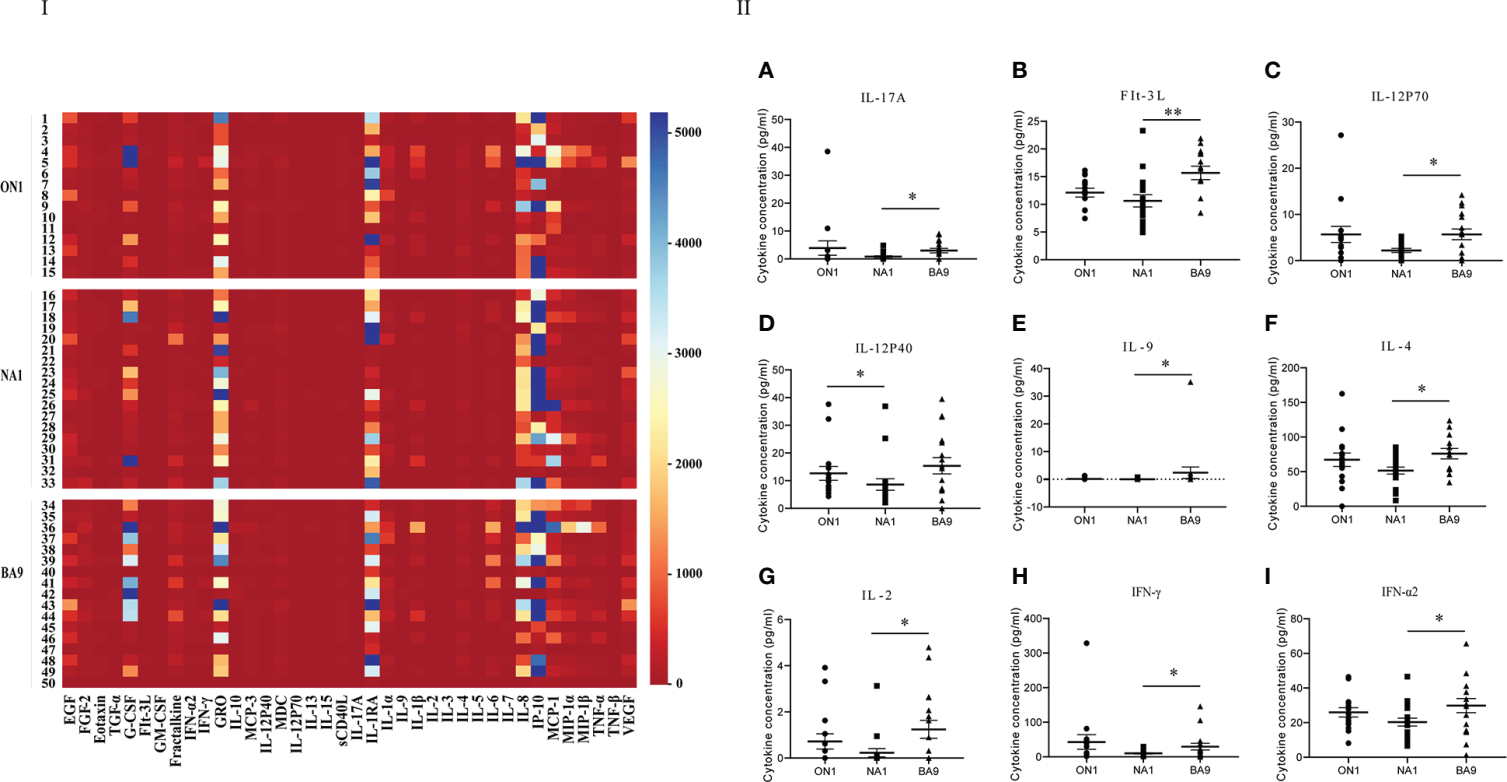
Comparison of cytokines and chemokines among respiratory specimens from patients infected with genotype ON1, NA1, or BA9 of RSV. I: the heat map. Each small square represents the specific value (pg/mL) of the cytokines and chemokines of each child. Different colors indicated the range of values of cytokines. For example, purple indicated the highest value, and red was the lowest. II: Significant differences were shown in the level of cytokines and chemokines (**A**: IL-17A; **B**: FIt-3L; **C**: IL-12P70; **D**: IL-12P40; **E**: IL-9; **F**: IL-4; **G**: IL-2; **H**: IFN-γ; I: IFN-α2) among respiratory samples from patients infected by genotype ON1, NA1, or BA9 of RSV. Each spot in the map represented the value (pg/mL) of the cytokines and chemokines of each patient. ●: samples from patients infected with genotype ON1; ■: samples from patients infected with genotype NA1; ▲: samples from patients infected with genotype BA9; *: significant differences were shown between the two groups (*P*<0.05). **: significant differences were shown between the two groups (*P*<0.01).

The values of cytokines and chemokines in the respiratory specimens from patients were shown in **Figure 2** of the heat map, in which GM-CSF, GRO, IL-1RA, IL-8, IFN-γ-induced IP-10, and MCP-1 were widely detected in all patients. The values of cytokines, GM-CSF, and the soluble cluster of differentiation 40 ligands (sCD40L), markers of innate immunity, IFN-α2, IL-1α, IL-1β, IL-2, IL-12p40 and IL-12p70, indicators of the T helper 1 (Th1) cell response, IL-4, IL-5, and IL-13, indicators of the Th2 response, and IL-17A, an indicator of the Th17 response, in patients infected by RSV NA1 were lower than those with ON1 or BA9. Significantly differences (t=-2.136, *P*<0.05) were shown in the level of IL-12p40 between patients with NA1 and ON1 and IFN-α2 (t=-2.091, *P*<0.05), IFN-γ (t=-2.013, *P*<0.05), IL-12p70 (t=-2.179, *P*<0.05), IL-2 (t=-2.339, *P*<0.05), IL-4 (t=-0.386, *P*<0.05), IL-9 (t=-1.96, *P*<0.05), IL-17A (t=-1.995, *P*<0.05), and FMS-like tyrosine kinase 3 ligand (Flt-3l) (t=-0.296, *P*<0.05) between patients with NA1 and BA9 (**Figure 2**).

**Figure 3:**
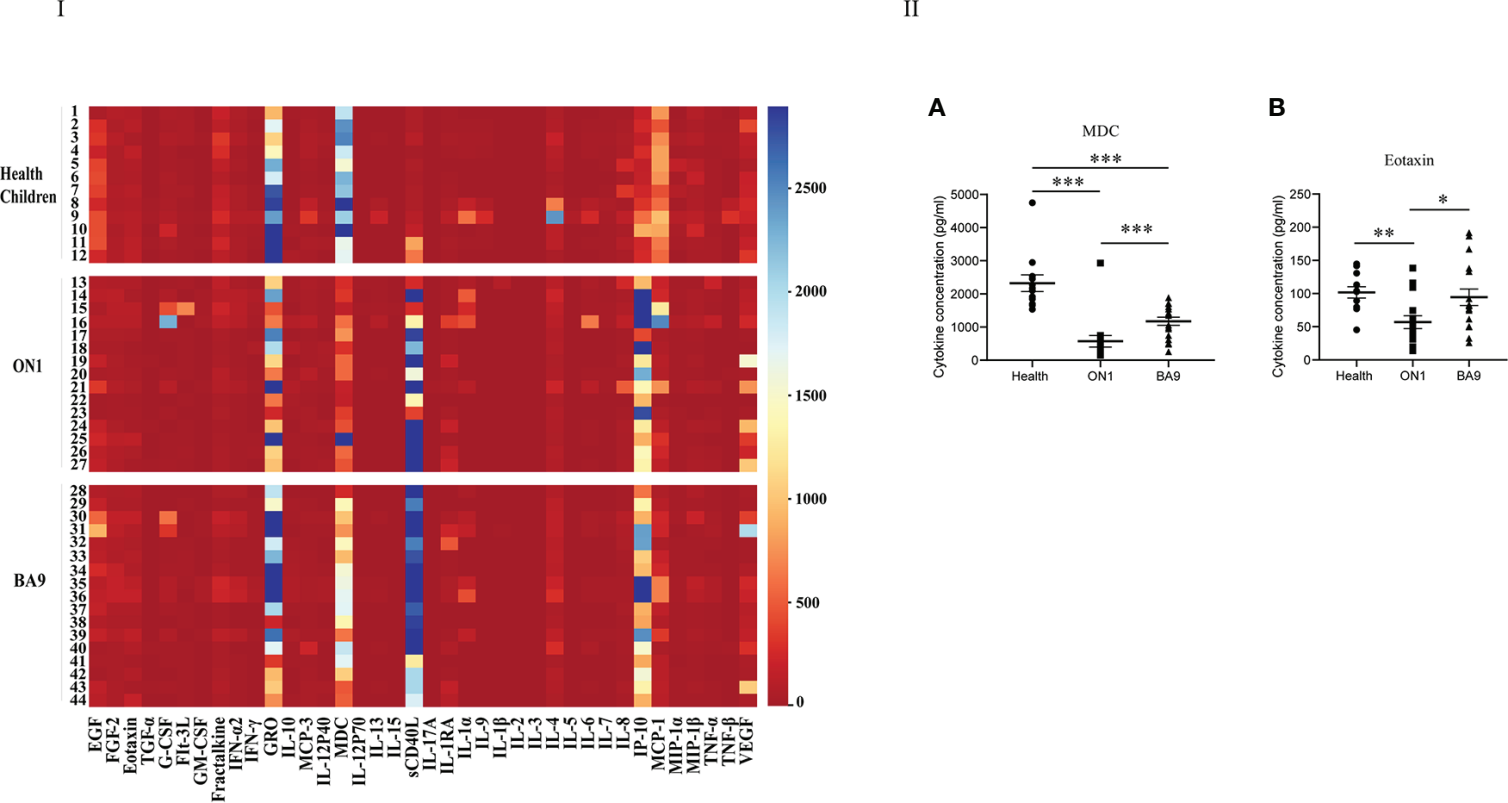
Comparison of cytokines and chemokines among the first serum samples from patients infected with genotype ON1, BA9 of RSV, or healthy children. I: the heat map. Each small square in the map represented the specific value (pg/mL) of the cytokines and chemokines of each child. Different colors indicated the range of values of cytokines. For example, purple indicated the highest value, and red was the lowest. II: Significant differences were shown in the level of cytokines and chemokines (A: MDC; B: Eotaxin) among the first serum group from patients infected by genotype ON1, BA9 of RSV, or healthy children. Each spot in the map represented the value (pg/mL) of the cytokines and chemokines of each child. ●: samples from healthy children; ■: samples from patients infected by genotype ON1; ▲: samples from patients infected by genotype BA9; *: significant differences were shown between the two groups (P<0.05). **: significant differences were shown between the two groups (*P*<0.01). ***: significant differences were shown between the two groups (*P*<0.001).

In the first serum group collected from patients of days 0 to 3 after the onset of the disease, GM-CSF, GRO, IL-1RA, IL-8, IP-10, and MCP-1 were different among patients shown in **Figure 3** of the heat map. In the scatter plot, only eotaxin (t=-0.86, *P*<0.05) and MDC (t=-3.342, *P*<0.05) were significantly different between patients with ON1 and those with BA9 (**Figure 3**). The eotaxin level in healthy children was significantly higher than in patients with ON1 but not significantly different from those with BA9. However, the level of MDC was significantly lower in both ON1 and BA9 groups than in healthy children.

**Figure 4:**
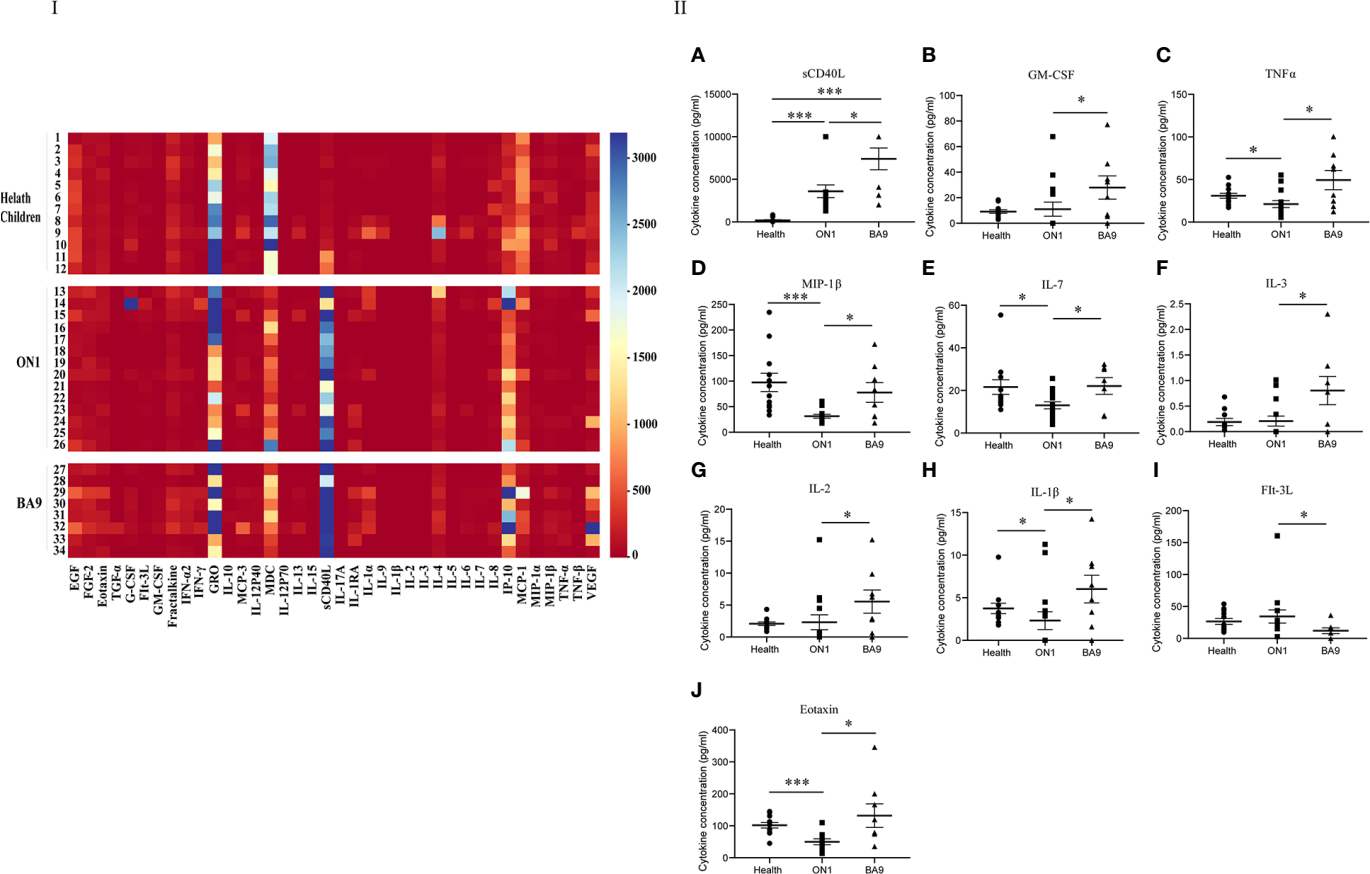
Comparison of cytokines and chemokines in the second serum group from patients infected with genotype ON1, BA9 of RSV, or healthy children. I: the heat map. Each small square in the heat map represented the specific value (pg/mL) of the cytokines and chemokines of each child. Different colors indicated the range of values of cytokines. For example, purple indicated the highest value, and red was the lowest. II: Significant differences were shown in the level of cytokines and chemokines (**A**: sCD40L; **B**: GM-CSF; **C**: TNFα; **D**: MIP-1β; **E**: IL-7; **F**: IL-3; **G**: IL-2; **H**: IL-1β; **I**: FIt-3L; **J**: Eotaxin) among the second serum group from patients infected with genotype ON1, BA9 of RSV, or healthy children. Each spot in the map represented the value (pg/mL) of the cytokines and chemokines of each patient. ●: samples from healthy children; ■: samples from patients infected by genotype ON1; ▲: samples from patients infected by genotype BA9; *: significant differences were shown between the two groups (*P*<0.05). ***: significant differences were shown between the two groups (*P*<0.001).

For the second serum group collected from patients on days 5 to 7 after the onset of the disease, the levels of GRO, sCD40L, IP-10, and MCP-1 differed among patients (**Figure 4**). As shown in the scatter plot (**Figure 4**), the levels of cytokines sCD40L (t=-2.358, *P*<0.05), IL-7 (t=-1.644, *P*<0.05), MIP-1β (t=-2.862, *P*<0.05), GM-CSF (t=-2.188, *P*<0.05), eotaxin (t=-2.03, *P*<0.05), Flt-3L (t=-2.257, *P*<0.05) IL-2 (t=-2.151, *P*<0.05), TNF-α (t=-2.389, *P*<0.05), IL-1β (t=-2.114, *P*<0.05), and IL-3 (t=-2.237, *P*<0.05) were lower in patients with ON1 than those with BA9. GM-CSF, IL-2, IL-3, and Flt-3L levels did not differ significantly between healthy children and RSV-infected individuals. Eotaxin, IL-7, MIP-1β, and TNF-α levels were lower in RSV-infected individuals than in healthy children, but the level of sCD40L in patients with ON1 was significantly higher than that in healthy children.

Between the first and second groups of serum samples, the levels of fibroblast growth factor-2 (FGF-2) (t=-1.485, *P*<0.05), Flt-3L (t=-2.25, *P*<0.05), and MIP-1β (t=-0.493, *P*<0.05) were significantly different between patients with ON1 and BA9 (**Figure S6**), whereas the levels of cytokines GM-CSF (t=-2.102, *P*<0.05), IL-2 (t=-2.193, *P*<0.05), TNF-α (t=-2.155, *P*<0.05), and IL-17A (t=-2.136, *P*<0.05) were lower in the first group of serum samples compared to the second group in patients with BA9 (**Figure S7**). Positive correlations were found between IFNα and IL-12p70 (rs=0.650, *P*<0.05), IFNγ and IL-12p40 (rs=0.332, *P*<0.05), IL-17A and IL-6 (rs=0.208, *P*<0.05), IL-17A and IL-8 (rs=0.277, *P*<0.05), eotaxin and IL-5 (rs=0.325, *P*<0.05), and eotaxin and fractalkine (rs=0.033, *P*<0.05) (**Figure S8**).

## 4 Discussion

RSV has been recognized as the major viral agent causing lower respiratory tract infections (LRTIs) in infants and young children worldwide. The spatial folding, post-transcriptional modification, and antigenic processing of proteins in viral epitopes and regions beyond epitopes were altered due to genetic variation, and the structural and spatial conformation determined the immunity of B and T cells induced by viral infection. Therefore, immune cells that react with one conformation of a protein fragment may not be able to recognize the altered conformation of a similar fragment of the protein in the virus (19). The frequent emergence of novel genotypes of RSV may be the main consequence of immune evasion, which may be related to the severity of the disease, outbreak, and repeated infections.

The highest difference in viral nucleotide found between subtype A and B was shown in the G gene (66.2-68.5%), followed by the SH and F genes, which might explain the differences in the secondary and tertiary structures found in G, SH, and F proteins, in which G protein with an irregular curl and low bond energy is more prone to a conformational change, leading to highly variable immunogenicity (20). Furthermore, Genotypes ON1 and NA1 of RSV-A had similar 3D modeling structures in F and SH proteins but significant differences in G protein among genotypes ON1, NA1, and BA9 (RSV-B) (**Figure 1**). Accumulating evidence showed that RSV glycoprotein G has important immune modulatory effects during RSV infection, which may induce an exacerbated Th2 type cytokine expression and affect the antiviral response through the modulation of perforin and granzyme B expression (21). In addition, antibodies induced by the G protein are mainly involved in inhibiting viral replication and in immune modulation by interfering with the antibody-mediated neutralization response. The differences in G protein sequence and structure among different RSV genotypes indicate the differences in immune responses elicited by RSV *in vivo*, which may be a potential mechanism of viral immune evasion.

In recent years, ON1 with a 72nt-insertion has gradually become the dominant genotype of subtype A, while BA9 with a 60nt-insertion is the dominant genotype of subtype B (15, 22), suggesting that the G protein with the highest structural variation might be a marker of immune escaping from herd immunity (15, 23). Zhang et al. found 10 positive gene selection loci in subtype A and 4 in subtype B when the analysis of RSV strains was collected in Beijing from 2006 to 2016 (15). Most of these loci were within HVR2 of the G gene. Another specific positive selective locus, 274 of the ON1 genotype, was found to be related to the immune evasion of RSV (24). Pretorius et al. postulated that the widespread duplication insertion mutations in ON1 and BA9 might result from immune pressure (25). Thus, under positive selection pressure, the dominant NA1 genotype is eventually replaced by ON1 in subtype A of RSV (20, 25).

For F protein is the major target protein for neutralizing antibodies induced by natural infection, it has been shown that the variability of F protein enables viral escaping from neutralization by anti-RSV monoclonal antibodies (26). Indeed, Palivizumab has decreased RSV viral loads and RSV-associated immunopathology in mice (27, 28). Our results showed that Palivizumab exerted inhibitory effects on all subtypes or genotypes of RSV. However, Palivizumab had stronger inhibitory effects on the reference strain than on the isolated strains, suggesting that the mutations of isolated strains may evade the antiviral effects of Palivizumab. The results that strain 61397-ON1 (IC_50_ 0.938 μg/mL) was more sensitive to Palivizumab than 69438-NA1 (IC_50_ 1.875 μg/mL) revealed that the immune evade of ON1 was not solely dependent on mutations at antigenic site II of the F protein, for mutation N276S at antigenic site II was with N in strains long, A2, and 3047 (GA2) and S in strains 61397 (ON1), 69438 (NA1), CH18537 (GB2), 86673 (BA9), and 69104 (BA9). Therefore, although F proteins are relatively conserved among different subtypes and genotypes, minor differences in nucleotides still exist in the region, which might account for some differences in the efficiency of the host immune responses (29).

In this study, cross-reactions were found between subtypes and genotypes of RSV. However, the immune response to RSV was subtype or genotype-specific. Serum from patient 22190, positive for RSV NA1, exhibited the strongest inhibition to strain 5540 (NA1) (IC_50_, 1:468) and the weakest inhibition to 86673 (BA9) (IC_50_, 1:87). The serum from patient 93158 was positive for ON1 had the strongest inhibition to the strain 61397 (ON1) (IC_50_, 1:417), and the weakest activity to CH18537 (GB2) (IC_50_: 1:129). The serum from the patient (92991 positives for BA9) had the strongest inhibition to strain 86673 (BA9) (IC_50_, 1:276) and the weakest inhibition to the 61397 (ON1) (IC_50,_ 1:60). These results indicate that those differences of inhibition might be induced by gene mutations of G, other than F which were conserved among subtypes and genotypes, of clinical RSV strains (30, 31).

The upper respiratory tract is the initial spot where the innate immune response to RSV infection (32) is induced by immune cells recruited to recognize pathogens and produce cytokines, such as eotaxin, FGF-2, and fractalkine. The final elimination of the virus depends on both innate and subsequent adaptive immune responses. Specifically, during infection, CD4^+^ T lymphocytes can be activated by toll-like receptor signaling pathways. Other signaling pathways induce Th1, Th2, and Th17 or regulatory T cells (Tregs) to drive immune responses (33). The level of cytokines was different from sample to sample, and they had different expression patterns. Almost all cytokines and chemokines correlate positively with disease severity in RSV infection. High levels of chemokines secreted from the airway epithelium were found in this study, which might be involved in the recruitment and activation of immune cells (31), leading to varying degrees of immune response and subsequently affecting the severity of the disease.

Surfactant viral protein genes, host cell receptor genes, and Th1/Th2 genes are involved in RSV infection (34). In RSV infections, Th2-biased immunity is related to the enhanced severity of respiratory diseases (35), although some studies have revealed an imbalance of Th17/Treg responses (36). A balance between Th1 and Th2 immune responses is required for effective viral clearance (37). The levels of Th1 cytokines (IFN-α2, IL-1α, and IL-1β) in respiratory specimens in patients with NA1 were lower than those with ON1, which explained that children with NA1 are more likely to have longer hospitalization and a more frequent incidence of severe disease than those with ON1 (38). The higher expression of Th1 cytokines such as TNFα, IFNγ, IL-1α, and IL-1β in the first group of serum samples from patients with ON1 compared to those with BA9 may explain why patients with ON1 are more likely to show symptoms of fever (39). Those patients with ON1 who had lower Th1 cytokines IL-2 and TNFα in the second group serum samples may explain a more rapid recovery than those with BA9.

IL-3 and IL-33 are associated with the prognostic value of RSV infections; their overexpression suggests enhancing inflammatory response (37). IL-3 and IL-33 indicate the severity of the disease to a certain extent. Lower levels of IL-3 in respiratory specimens and the first and second group of serum samples in patients with ON1 compared to BA9 were found in this study, suggesting that patients with ON1 are more likely to have that milder disease (38).

During infection, the secretion of many cytokines shows two peaks: the early stage (days 1–3) and the late stage (days 6–8). The cytokines produced by T cells in the second peak may lead to the aggravation of RSV infection (38). There were significant differences in FGF-2, Flt-3L, and MIP-1β between the first and second serum samples in patients with ON1. These data suggest that innate immunity is the first line of defense in the early stage of infection. Lower Th1 and Th17 reactions in the first serum samples of patients with BA9 suggest that adaptive immunity is involved in the later stage of infection.

It has been suggested that the F protein of RSV mainly induces Th1-type responses and secretes IL-12 to induce IFN-α production, thereby facilitating Th1 cell differentiation (40), which is consistent with our data showing that IFNα and IL-12p70 are positively correlated. IL-12 and IFN-γ can promote the differentiation of Th cells into Th1 cell subsets. Antigen-presenting cells can secrete IL-12 after stimulation by IFN-γ and CD40L, which can induce natural killer cells to secrete a larger amount of IFN-γ, consistent with our data showing that IFNγ and IL-12p40 are positively correlated. IFN-γ can further enhance the expression of T-bet, which promotes the expression of IFN-γ itself in a positive feedback manner; this so-called “waterfall effect” can further promote the differentiation of T cells towards Th1 (41). In addition, there is also a positive correlation between eotaxin and fractalkine.

RSV G protein can induce CD4^+^ T cells, basophils, and monocytes to secrete Th2 cytokines (42). The later Th2 response can produce fractalkine and guide IL-5-activated eosinophils to traffic to the lungs, leading to the infiltration of eosinophils (43). Mice with RSV infection can produce Th2 cytokines (IL-4, IL-5, and IL-13), leading to airway remodeling and mucus overproduction (44, 45). It has been suggested that Th2 and Th17 responses induce allergic asthma by enhancing airway hyperresponsiveness, airway remodeling, and mucus production (20), which can promote the progression of RSV infectious diseases. IL-8, IFN-α, IL-6, TSLP, IL-3, and IL-33 are associated with the severity of RSV infections (37).

This study found a significant positive correlation between IL-17A and IL-6. IL-6 can activate Th17 lymphocytes by activating Th0 cells through the signal transducer and activator of the transcription 3 pathway. Th17 lymphocytes can recruit neutrophils and promote the development of lymphoid structures in the lungs, thereby leading to serious autoimmune inflammation (20). A significant positive correlation between IL-17A and immune cells was found in this study, which might result from the cross-talk and cooperation among IL-17, IL-1β, and TNF-α. Specifically, IL-17 may act on stromal cells to recruit neutrophils and promote the proliferation of fibroblasts and epithelial cells, among others. Then those cells may secrete inflammatory cytokines such as FGF-2, VEGF, GM-CSF, IL-8, eotaxin, MIP-1α, and MIP-1β to promote inflammation (46).

There were several limitations in this study. Due to the absence of NA1 strains in recent years, NA1 strains in this study were only collected from retrospective research from 2014 to 2015. Secondly, for the small sample size of clinical specimens used in the comparison of cytokines and chemokines, only “unpaired” in “Experimental design”, and “Use nonparametric test in “Assume Gaussian distribution” were used in statistical analysis. More data should be accumulated. However, the data collected in the study will be helpful for future studies when novel genotypes appear.

In conclusion, the immunology results indicated that the monoclonal antibody Palivizumab, specific to antigen site II of F, had stronger inhibition to subtype A than to subtype B and to reference strains than to isolated strains in Beijing. In contrast, RSV-positive human sera had higher inhibitory effects on the same origin subtype or genotype of RSV. The inductions of innate and adaptive immunity were diverse among different subtypes or genotypes of RSV, with higher adaptive responses in patients infected with genotype ON1. All these results may be explained by the viral genomic variation, including mutations in antigenic site II of F and the duplicate nucleotide insertions of G. The duplication insertions were found in ON1 and BA9. G protein had the highest variation, leading to substantial conformational and major immunogenicity changes. These results might explain why reinfection of RSV occurs and why there are differences in severity related to the subtype and genotype of RSV. These results provide useful references for developing antibodies, vaccines, and therapeutic drugs to treat RSV.

## Data availability statement

The datasets presented in this study can be found in online repositories. The names of the repository/repositories and accession number(s) can be found in the article/**Supplementary Material**.

## Ethics statement

The studies involving human participants were reviewed and approved by the Ethics Committee of the Capital Institute of Pediatrics (SHERLLM2015012). Written informed consent to participate in this study was provided by the participants’ legal guardian/next of kin.

## Author contributions

LZ conceived and designed the experiments. XZ, MJ, FJW, and YX performed the experiments. LZ, YQ, and RD analyzed the data. XZ, FW, YS, and RZ handled clinical samples and isolated viruses. XZ, MJ, F(J)W, YX, and RD wrote the paper. LZ and RD reviewed and revised the manuscript. QS, DQ, LC, and LM collected clinical data and samples. All authors contributed to the article and approved the submitted version.

## Supplementary material

Supplementary Figure 1 Maximum likelihood phylogenetic trees of the HVR2 region in RSV G, in which subtypes A and B and different genotypes of RSV were identified. Sequences obtained from the study were labeled with black dots. Others were sequences downloaded from GenBank.

Supplementary Figure 2 Homology and variability analysis of nucleotide and amino acid shown in A and B, respectively, between subtype A and B; in C and D, respectively, between genotype ON1 and NA1 of RSV-A.

Supplementary Figure 3 72nt-insertions in HVR2 of G in 61397 (ON1) of RSV.

Supplementary Figure 4 60nt-insertions in HVR2 of G in 86673 (BA9) and 69104 (BA9) of RSV

Supplementary Figure 5 Secondary structures of surface glycoproteins of RSV ON1, NA1 and BA9, including SH of 61397-ON1 **(A)**, 69438-NA1 **(B)**, 86673-BA9 **(C)**, G of 61397-ON1 **(D)**, 69438-NA1 **(E)**, 86673-BA9 **(F)**, and F of 61397-ON1 **(G)**, 69438-NA1 **(H)**, and 86673-BA9 **(I)**.

Supplementary Figure 6 Maximum likelihood phylogenetic trees of the HVR2 region in RSV G for the subtyping of respiratory specimens collected to evaluate immune response. Sequences obtained from the study were labeled with black dots. Others were sequences downloaded from GenBank.

Supplementary Figure 7 Cytokines showed a significant difference between the first and second serum groups from patients positive for RSV ON1.

Supplementary Figure 8 Cytokines showed a significant difference between the first and second serum groups from patients positive for RSV BA9.

Supplementary Figure 9 Cytokines showed positive pairwise correlations among patients with RSV.

Supplementary Table 1 Primers designed for genomic sequencing of RSV.

Supplementary Table 2 Homology and divergence analysis of whole genomic sequences from isolates of RSV.

Supplementary Table 3 Physicochemical properties of F, G, and SH of RSV dominant genotypes ON1, NA1, and BA9 were analyzed by ProtParam.
