# Supplementary material for: Immune escaping of the novel genotypes of human respiratory syncytial virus based on gene sequence variation
Source: Front Immunol. 2023 Jan 10;13:1084139. doi: 10.3389/fimmu.2022.1084139 (PMC9871593; doi:10.3389/fimmu.2022.1084139)
Supplement: Supplementary Figure 1 — Maximum likelihood phylogenetic trees of the HVR2 region in RSV G, in which subtypes A and B and different genotypes of RSV were identified. Sequences obtained from the study were labeled with black dots. Others were sequences downloaded from GenBank. [file DataSheet_1.pdf]

Supplementary Material

Supplementary Figures

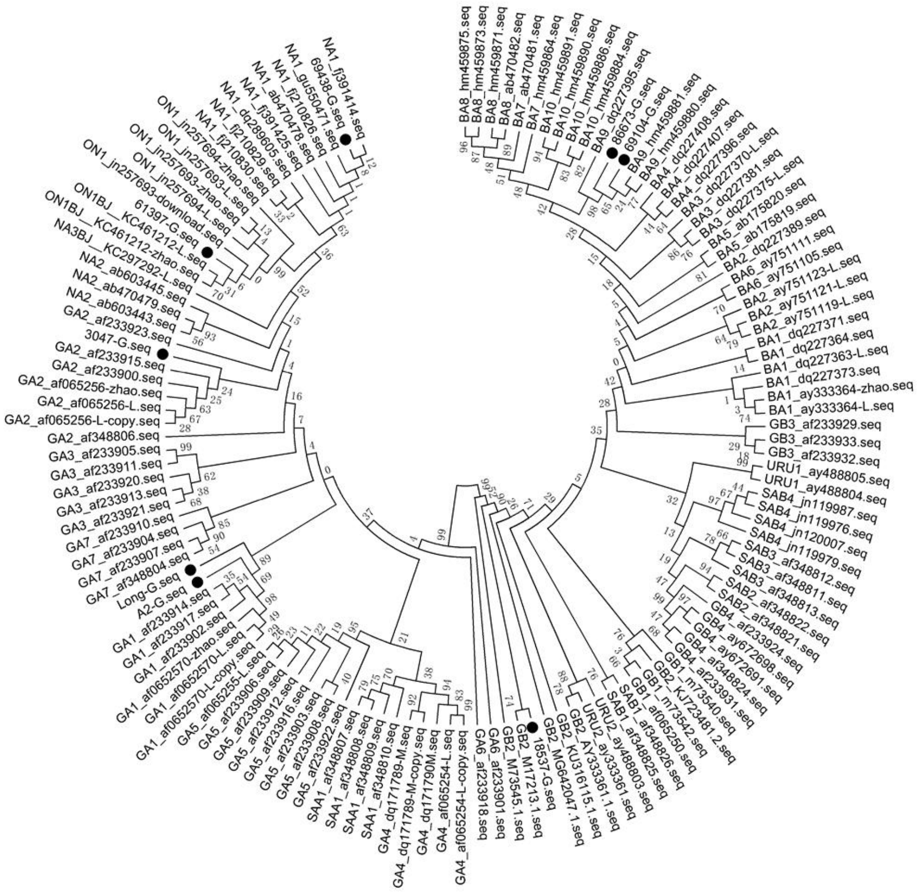

Figure S1

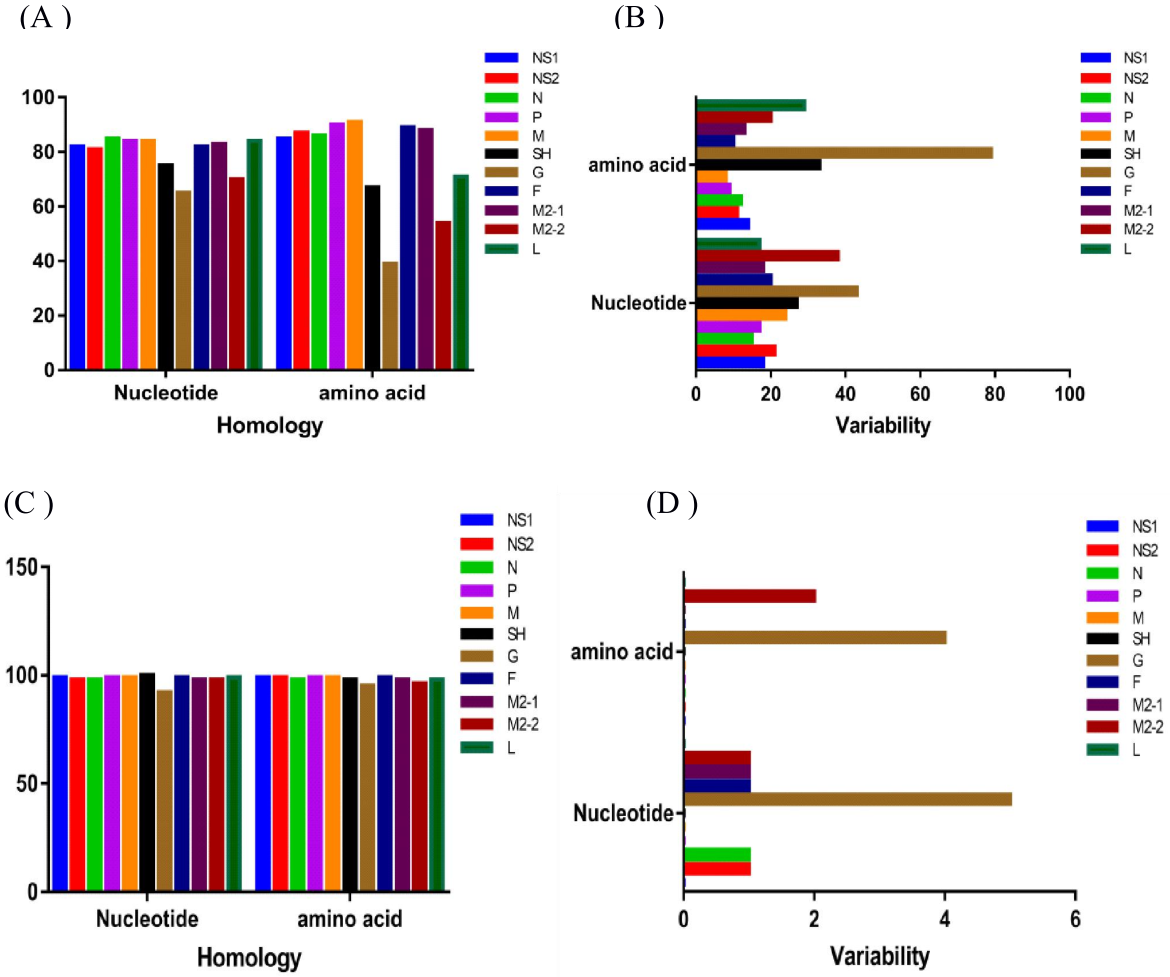

Figure S2

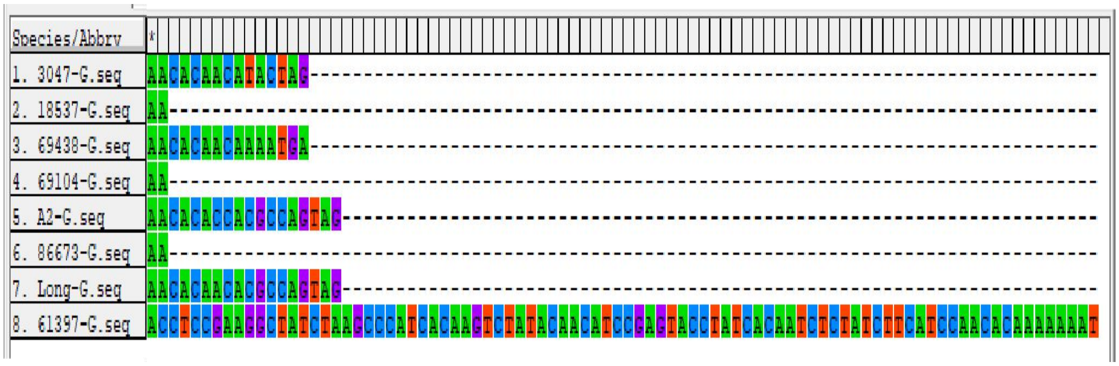

Figure S3

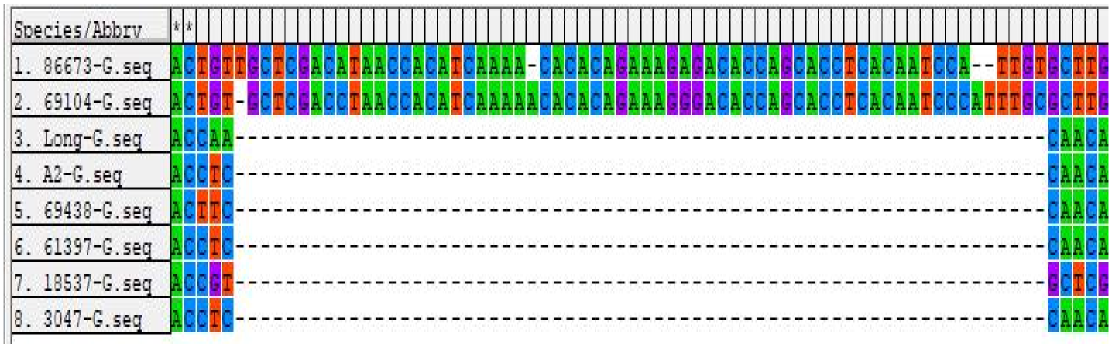

Figure S4

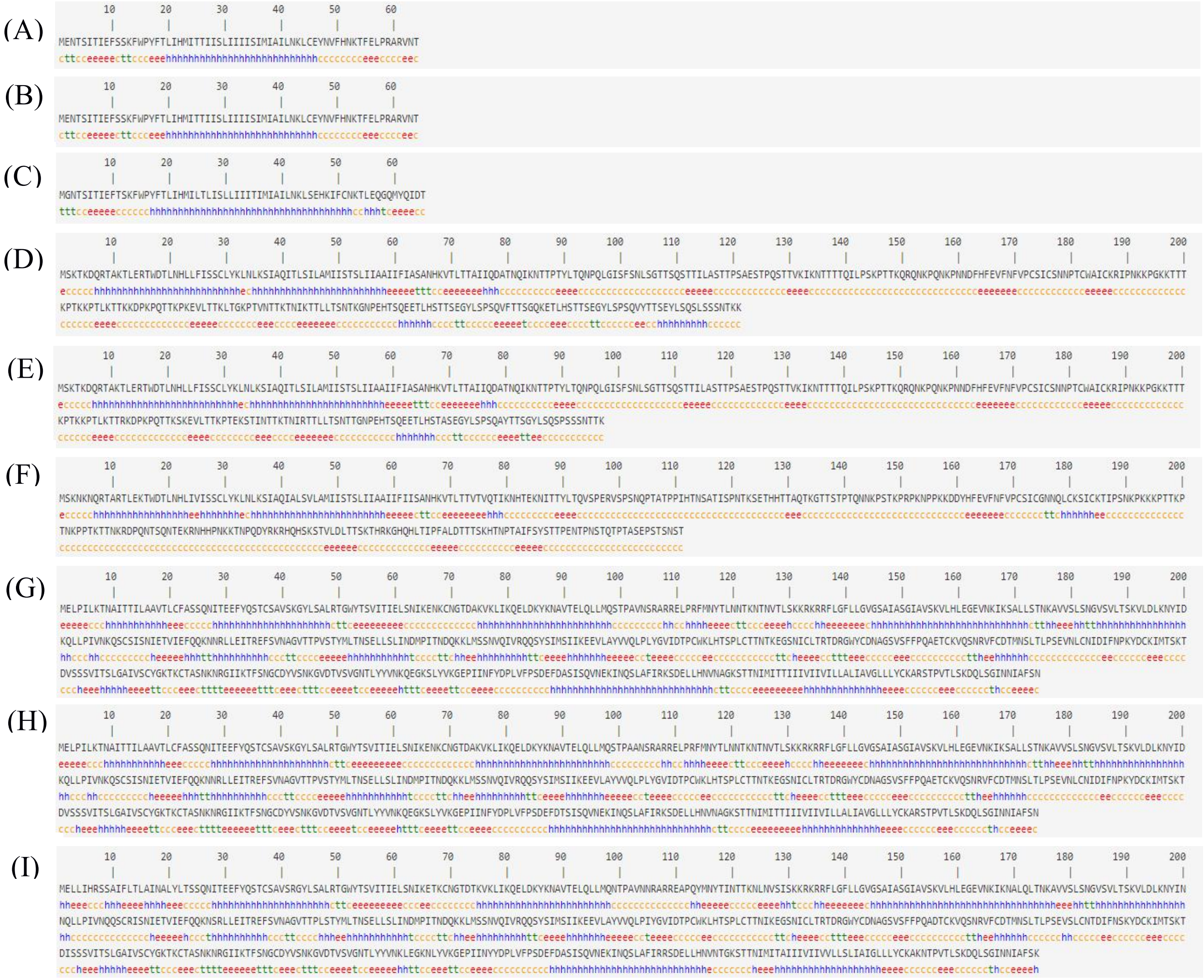

Figure S5

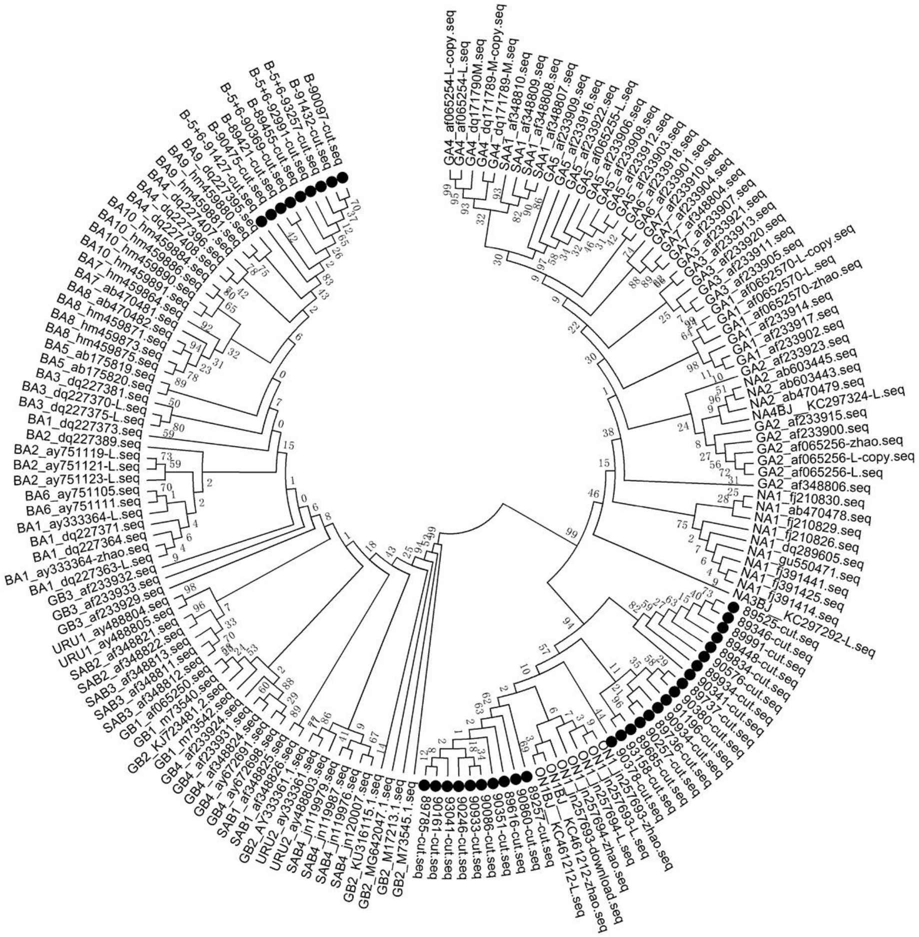

Figure S6

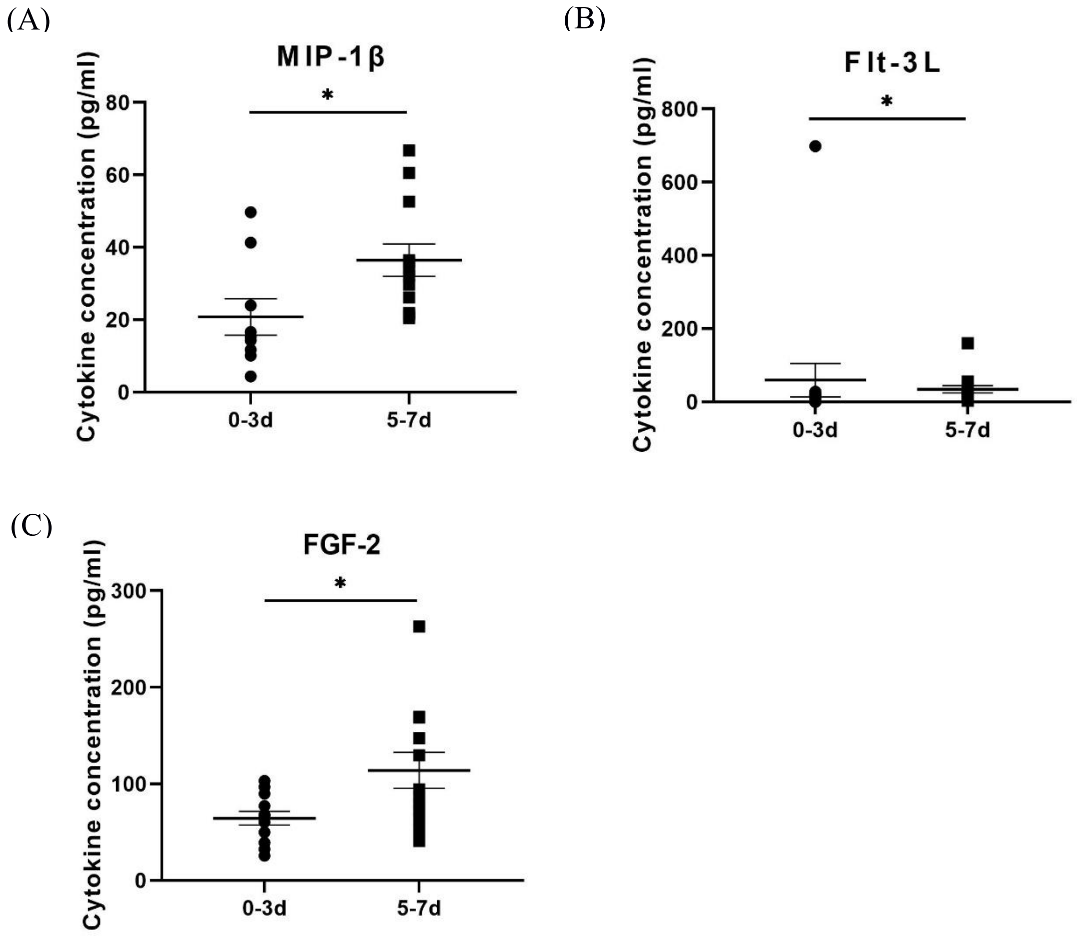
(A)
MIP-1β
Cytokine concentration (pg/ml)
80
60
40
20
0
*
0-3d
5-7d
(B)
Flt-3L
Cytokine concentration (pg/ml)
800
600
400
200
0
*
0-3d
5-7d
(C)
FGF-2
Cytokine concentration (pg/ml)
300
200
100
0
*
0-3d
5-7d

Figure S7

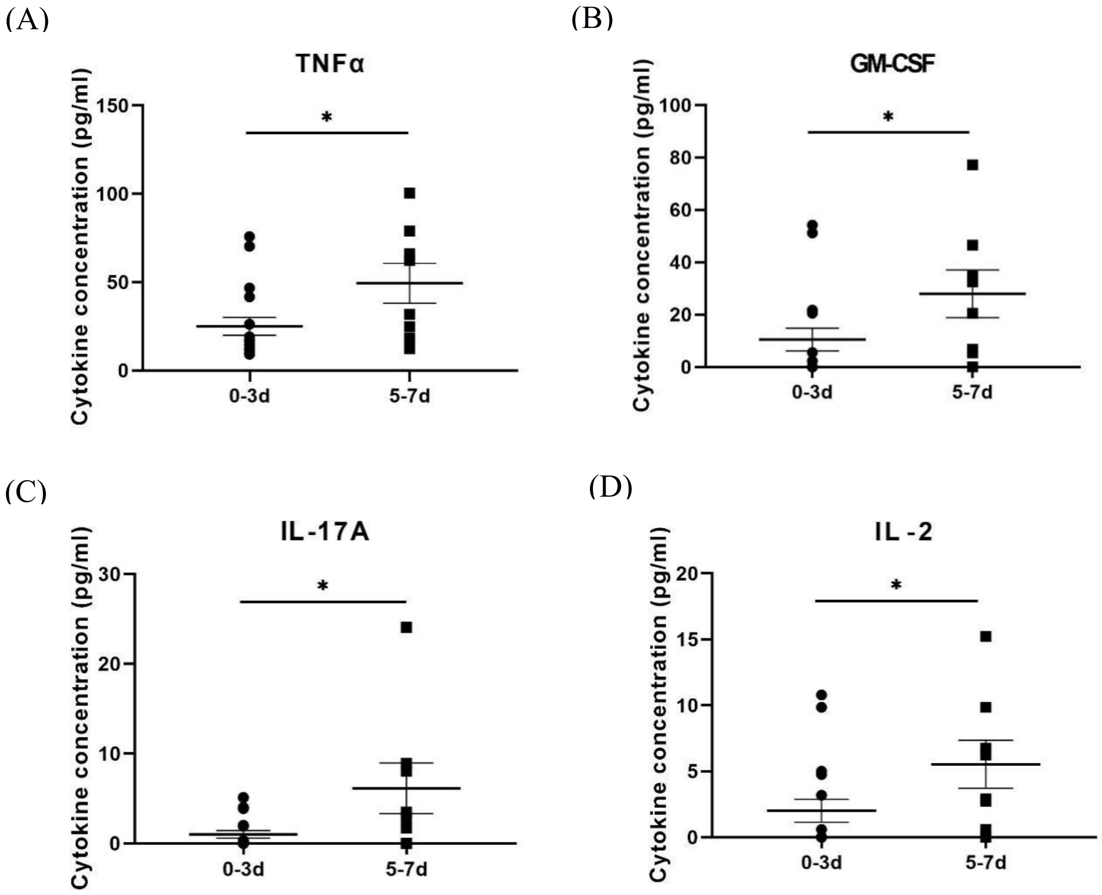
(A)
TNFα
Cytokine concentration (pg/ml)
150
100
50
0
*
0-3d
5-7d
(B)
GM-CSF
Cytokine concentration (pg/ml)
100
80
60
40
20
0
*
0-3d
5-7d
(C)
IL-17A
Cytokine concentration (pg/ml)
30
20
10
0
*
0-3d
5-7d
(D)
IL-2
Cytokine concentration (pg/ml)
20
15
10
5
0
*
0-3d
5-7d

Figure S8

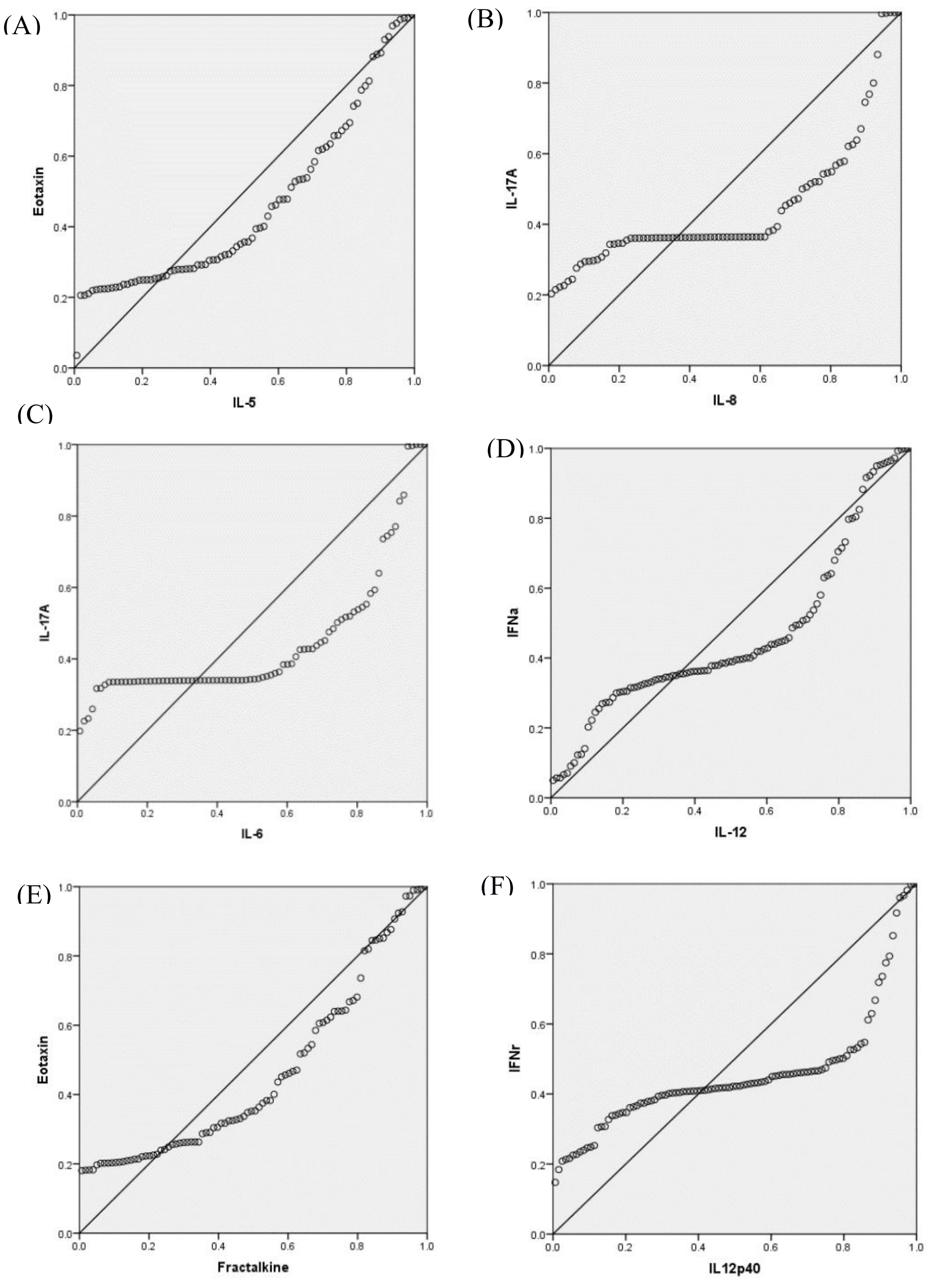
(A)
Eotaxin
IL-5
(B)
IL-17A
IL-8
(C)
IL-17A
IL-6
(D)
IFNa
IL-12
(E)
Eotaxin
Fractalkine
(F)
IFNr
IL12p40

Figure S9

Supplementary Tables

Table S1. Primers designed for genomic sequencing of RSV

Primers	**Sequences（5'-3'）**
hRSV 1F	GTAAAACGACGGCCAGACGCGAAAAAATGCGTACAAC
hRSV 1R	TGGATGGTGTATTTGCTGG
hRSV 1F-2*	ACGCGAAAAAATGCGT
hRSV 1R-2*	GTTGGATGGTGTATTTGCT
hRSV 2F	ATATCAAACCAAKAKTCAAAC
hRSV 2R	TTCAGGAGCAAACTTTTCCAT
hRSV 2F-2 *	CATTGGCATTAAGCCTAC
hRSV 2R-3*	TGTTTGCATCTTCTCCATG
hRSV 2R-B*	GCTTAGTGTAACTGGTGT
hRSV 3F	AGAAATTGGGTGGWGAAGC
hRSV 3R	CCCTTGGGTGTGGATATTTG
hRSV 4F	AACCTRTTGGAAGGGAATG
hRSV 4R	AGGCCAGAATTTGCTTGAG
hRSV 5F	ACMAACMCTCTGTGGTTC
hRSV 5R*	GCTTGGTGGTGGTTTTCTTTCCAGG
hRSV 5F-B*	CAATGATAATCTCAACCTCTC
hRSV 5F-2	CACACTGGAAGCAYACAGCCACAC
hRSV 5R-B	CACGGTTCTCTGCTAAGGT
hRSV 5F-3	TTGGTTTATTGCTTGGTAT
hRSV 6F	GAGTCAACCCCACAATCCAC
hRSV 6R	GCATTAACACTAAATTCCCTGGT
hRSV 6R-B*	ACTTTAGTGTCAGTTCCATTG
hRSV 6R-B2*	GATCAGCAACTCCATGG
hRSV 7F	TGCACCTAGAAGGGGAAGTG
hRSV 7R*	TAATGTGACTGGTGTGCTTCTGGC
hRSV 7F-2*	GGAGAAGTGAACAAGATC
hRSV 7R-3*	CTTAGTGTRACTGGTGTG
hRSV 8F	CCCATTAGTRTTCCCCTCTG
hRSV 8R	TCCATTAATAATGGGATCCATT
hRSV 9F	TGCCAGCAGACGTATTGAAG
hRSV 9R	TTTATTATGTAGAACCCCTCATTGTG
hRSV 10F	CAATGCAACATCCTCCATC
hRSV 10R	GGTTGCATTGCAAACATTCT

hRSV 11F	CGTGAGTTTCGGTTGCCT
hRSV 11R	GGGATCACCACCACCAAAT
hRSV 12F	AGTGGGACCGTGGATAAAC
hRSV 12R	TGACTGTAAGGCGATGCAAATAG
hRSV 13F	TGGACATCAAATATACWACAAGC
hRSV 13R	TTAACAACCCAAGGGCAAAC
hRSV 14F	AAAAAGATTGGGGAGAGGGAT
hRSV 14R	TGCAYTTTCTTACATGCTTGC
hRSV 15F	GGTGAAGGAGCAGGGAATTT
hRSV 15R	CAGGAAACAGCTATGACACGAGAAAAAAAGTGTCAAAAACT
hRSV 15F-2*	TGTATAGCATTCATAGGTG
hRSV 15R-A*	GAAAAAAAGTGTCAAAAAC
hRSV 15R-B*	CCCTTAAGACTAAAATGAT

* refer to the RSV genome sequences of RSV A strain ATCC VR26 (AY911262) and RSV B strain (KF826843), downloaded from GenBank and designed by Primer Premier 5.0 software. The remaining primers were referred to Kumaria et al.

Table S2. Homology and divergence analysis of whole genomic sequences from isolates of RSV

Homology (%)

		**RSV-A**					**RSV-B**		
		**GA2 (3047)**	**ON1 (61397)**	**NA1 (69438)**	**GA1 (A2)**	**GA1 (Long)**	**GB2 (CH18537)**	**BA9 (86673)**	**BA9 (69104)**
**RSV-A**	**3047**		96.9	96.9	95.3	95.4	81.0	81.0	80.9
	**61397**	3.1		98.8	94.6	94.7	80.9	80.6	80.5
	**69438**	3.1	1.2		94.6	94.7	80.8	80.7	80.6
	**A2**	4.7	5.4	5.4		98.1	81.3	81.2	81.2
	**Long**	4.6	5.3	5.3	1.9		81.3	81.1	81.1
**RSV-B**	**CH18537**	19	19.1	19.2	18.7	18.7		95.9	96.1
	**86673**	19	19.4	19.3	18.8	18.9	4.1		99.3
	**69104**	19.1	19.5	19.4	18.8	18.9	3.9	0.7	

Divergence (%)

Table S3. Physicochemical properties of F, G and SH of hRSV dominant genotypes ON1, NA1 and BA9 analyzed by ProtParam

**Surface glycoproteins**	**ON1**			**NA1**			**BA9**		
	SH	G	F	SH	G	F	SH	G	F
**Number of amino acids**	64	321	574	64	297	574	65	311	574
**Molecular weight**	7535.99	35235.12	63333.18	7535.99	32573.05	63335.15	7579.11	34555.06	63676.46
**Theoretical pI**	7.94	9.87	9.06	7.94	9.95	9.06	6.7	10.1	9.08
**Dominant amino acids**	Ile 20.3%<br>Thr10.9%<br>Asn 7.8%	Thr 19.6%<br>Lys 10.9%<br>Leu 10.3%	Ser 10.5%<br>Leu 10.3%<br>Thr 8.5%	Ile 20.3%<br>Thr10.9%<br>Asn 7.8%	Thr 19.9%<br>Ser 11.4%<br>Lys 10.1%	Ser 10.5%<br>Leu 10.3%<br>Thr 8.7%	Ile 20.0%<br>Leu12.3%<br>Thr12.3%	Thr 18.0%<br>Lys 10.6%<br>Ser 10.0%	Ser 10.5%<br>Leu 10.5%<br>Asn 8.7%
**Instability index (<40 stable, >40 unstable)**	35.54	30.31	41.82	35.54	32.81	41.56	36.23	28.62	41.18
